# The Army Medical Service

**Published:** 1923-09

**Authors:** 


### THE ARMY MEDICAL SERVICE.

**BY A MAN IN RETIREMENT.**

THE Editor has asked me, a man in retirement, to write on the work of "an Army doctor." He says "No doubt it has its light as well as its serious side." That sentence I regard as a hint that he does not want description of the trodden path, but prefers to publish something of the interests of the Army doctor's life outside its routine.

### What Life is For.

What is the object of life? It is an old question. Here is an answer, right or wrong. The object of life is to live and be happy. That is how I read the revelations of the universe to be found by watching the ways of the creatures in it. It is a simple philosophy until the question comes—what is to be happy? Every one then has his own ideas. However, no man is unhappy who has a good digestion and enough to digest. The first comes of hard exercise and temperate use of meat and drink; the last can be earned in the R.A.M.C. The career will lead only to a sufficiency of the good things of life. Great wealth will not accrue. Often the growl comes, "Look at me and look at So-and-so! He was two years junior to me at the hospital and now he is doing two thousand a year."

### The Contrast.

True, my friend—but what are So-and-so's expenses and anxieties and what are yours? What is So-and-so's life and what is yours? If you wanted a fixed abiding place, a domestic hearth and steady grind in the same suburb, year after year, it was yours to take, if you had the wherewithal to buy into a practice with a sufficient basis of panel and appointments. But you cannot expect to go into the Army and settle to a quiet life. If you did expect that you must have joined up without enquiry. It is ever the grumble of the "disgruntled" that thay are moved so often. Make no mistake, you who would come in. You must be ready to live in your boxes. If you want a wife and family and the calm domestic hearth before you are thirty, the Army is not your place. Make up your mind what you want before you take the shilling, and if you want the quiet delights of general practice don't touch the coin. Don't imagine you are going to have a life all play and no work. There is much work to do, and you will not be allowed to be happy if you do not do the job allotted to you properly and without shirking. If you have zeal for research, it is a good career to be an Army doctor, for in no other can a man indulge a passion for pathology and make as good an income as his colleague who doses the babies and canoodles their mothers.

### Peripatetics and Leave,

An officer of the R A.M.C. experiences many shifts of station, sometimes several in a year. His compensation is that he can see the world, if he wishes. Unfortunately, Canada and South Africa no longer require the services of the Corps; but India, from Cape Comorin to Peshawar, the West Indies, China, Palestine, the Soudan, to name only some, are open to him, and he travels free of charge unless he overloads himself with impedimenta. It is not possible for every officer to have his leave exactly when it is most convenient to him. Nevertheless, leave comes in good slices, if it is asked for properly and taken when opportunity serves. Then is the chance to visit scenes and places worth the visiting. Where to go is a matter of taste. I knew a brother officer who visited in course of years a large part of the more wonderful temples of India. The taste of another often took him in queer boats with slant-eyed crews up hundreds of miles of Chinese rivers. He himself preferred to all other remote and wild places in the Himalayas and the jungles, in which the enjoyment of magnificent scenery can be combined with some sport with the rifle. Heads of barasingha, burrell, thar, gooral, and the skins of bears, now that the time of retirement is come, conjure up memories of Cashmere, Chamba, Pangi, Lahool, Kumaon and Ghurwal, breakneck climbs, tremendous precipices, spacious fields of ice and snow; dark forests of pine above tropical valleys; little camps perched high on narrow ledges of broad green mountain sides; shikaris and coolies; hospitable village head men; visits with their priests to carven images in lonely shrines and offerings to idols. Other heads and skins, sambur and spotted deer, tiger and panther bring back to the memory great forests, exhausting heat, the stealthy tread of stags down the glades, the rasping grunting of leopards, peacocks spreading gorgeous fans before their sober-clad harems, the chatter of monkeys, the screech of parrots, the thousand noises and silences of the jungle.

### The Thrills of Pigsticking.

Pigsticking and horses for it are within the means of the bachelor officer of the R.A.M.C. It is one of the finest sports of the world both for picturesqueness and excitement. The great sea of grass spread along the banks of the river, the long line of dusky beaters with here a camel carrying soda-water, there a pad elephant, the rush of startled buck, the sight of slinking jackals and creeping cats moving before the line, the sudden yells of "*Soor! Soor!*" as a good boar goes away. Then the race for first spear, the jinks, the croppers, and the end a great fight with a wounded, charging boar; camp beneath the mango's shade, dinner in the moonlight, and after it cheroots and endless yarns of horses and shikar.

### Sport or Wife?

It is good to have stores of such memories. They are not for those who spend their Indian leave in hill hotels. For a junior officer of sporting tastes and free from ties, the R.A.M.C. is a pleasant service enough. His pay will suffice for life in a comfortable mess, for any game he likes to play, and for much good sport if he be so inclined. If he have some income beyond his pay, so much the better for him. He must not "grouse," though, because he has not pay enough to support a wife and family in comfort and also to indulge in the pastimes of the free. It is not reasonable. Altruism is its own reward. A sacrifice that is not felt is no sacrifice at all. Still, there is pathos in the old Indian advertisement, "For sale, a double-barrelled gun and two polo ponies. Will take in part exchange a two-seated perambulator and a croquet set."

### Pelf and Pension.

The work is interesting and there is much of it. The financial reward is comparable to a practice bought at £700 a year, worked up to £1,500 a year and sold for retirement on the price got and some few savings. The pension earned is worth about ten years' purchase of its annual value at 55 years of age. Consider well if the life will suit before you elect to live it, and having chosen take cheerfully the rough with the smooth. Each man must decide for himself what he prefers to do. Is it better to put down £1,000 for a good practice, marry early, settle down for life, and work up the practice to £1,500 or so, or to join the corps, delay marriage till the forties, knock about the world, and take the fun in life the corps gives ? It is a matter of taste, but a man cannot, as the slang expression goes, "have it both ways."

